# Towards the Establishment of a Sea Urchin Sperm Biobank: Key Challenges and Opportunities for the Conservation and Sustainable Aquaculture of *Paracentrotus lividus*

**DOI:** 10.3390/ani16172788

**Published:** 2026-09-04

**Authors:** Emanuele Antenucci, Michele Di Iorio, Celeste Santoianni, Amedea Mastronardi, Martina Cofelice, Francesco Lopez, Sebastiano Rosati, Lucia Maiuro, Elena Sorrentino, Giulia Carone, Nicola Zizzo, Yusuf Bozkurt, Alessandra Roncarati, Nicolaia Iaffaldano

**Affiliations:** 1 Department of Agricultural, Environmental and Food Sciences, University of Molise, Via De Sanctis snc, 86100 Campobasso, Italy; emanuele.antenucci@unimol.it (E.A.); michele.diiorio@unimol.it (M.D.I.); celeste.santoianni@unimol.it (C.S.); amedea.mastronardi@unimol.it (A.M.); martina.cofelice@unimol.it (M.C.); lopez@unimol.it (F.L.); or sebastiano.rosati@unito.it (S.R.); maiuro@unimol.it (L.M.); sorrentino@unimol.it (E.S.); 2 Department of Agricultural, Forestry and Food Sciences (DISAFA), University of Turin, Largo Paolo Braccini 2, 10095 Grugliasco, Italy; 3 Department of Veterinary Medicine, University of Bari Aldo Moro, Valenzano, 70010 Bari, Italy; g.carone26@studenti.uniba.it (G.C.); nicola.zizzo@uniba.it (N.Z.); 4 Department of Aquaculture, Faculty of Fisheries, Mersin University, 33160 Yenişehir, Mersin, Türkiye; yusuf.bozkurt@mersin.edu.tr; 5 School of Biosciences and Veterinary Medicine, University of Camerino, Viale Circonvallazione 93–95, 62024 Matelica, Italy; alessandra.roncarati@unicam.it

**Keywords:** *Paracentrotus lividus*, conservation aquaculture, integrated multi-trophic aquaculture (IMTA), sperm cryopreservation, germplasm biobank, AVATIM project

## Abstract

The Mediterranean Sea urchin *Paracentrotus lividus* is a key benthic herbivore and a valuable fishery resource because of the high commercial and gastronomic value of its edible gonads. Increasing market demand, overharvesting, climate change, habitat degradation, and pollution have contributed to the decline of wild populations in several Mediterranean areas. This narrative review, including a preliminary case study, provides an integrated perspective on the role of *P. lividus* in conservation-oriented aquaculture, with particular attention to Integrated Multi-Trophic Aquaculture (IMTA), microbiological quality, sperm quality assessment, cryopreservation, and germplasm biobanking. IMTA may improve nutrient recycling and resource-use efficiency, whereas cryopreservation and biobanking can support genetic resource conservation, selective breeding, hatchery production, and future restocking programs. Within this framework, the ongoing AVATIM project (“Virtuous Aquaculture in Integrated Marine Multitrophic Aquaculture”) is presented as an operational case study illustrating how reproductive biotechnology may be integrated into sustainable multitrophic aquaculture and germplasm conservation strategies. Baseline sperm quality parameters of sexually mature males collected from the southern Adriatic Sea were assessed using Computer-Assisted Sperm Analysis and flow cytometry, providing baseline values for future cryopreservation studies and biobanking applications. Overall, AVATIM illustrates how IMTA, reproductive biotechnology, and genetic resource conservation may jointly support sustainable Mediterranean Sea urchin management.

## Simple Summary

Natural populations of the Mediterranean Sea urchin *Paracentrotus lividus* are declining in several coastal areas due to overfishing, habitat degradation, pollution, and climate change, while demand for its edible gonads remains high. This narrative review, including a preliminary AVATIM case study, summarizes current knowledge on the ecological and economic relevance of *P. lividus* and its potential role in conservation aquaculture and Integrated Multi-Trophic Aquaculture (IMTA). It also highlights the importance of sperm quality assessment, cryopreservation, and germplasm biobanking for sustainable management. Within this framework, the AVATIM project provides baseline reproductive and sperm quality data for future cryobanking applications. The results indicate high sperm motility and viability, providing baseline information for future cryopreservation and germplasm preservation studies. Overall, IMTA and biobanking may represent complementary tools to reduce pressure on wild populations, support hatchery production, preserve genetic resources, and contribute to more sustainable and resilient Mediterranean aquaculture systems.

## 1. Introduction

In recent years, growing global demand for seafood and the intensification of human activities in coastal ecosystems have led to a progressive increase in pressure on natural marine resources [1,2]. At the same time, the effects of climate change, pollution, and habitat degradation are profoundly altering the structure and functionality of marine ecosystems, making the adoption of sustainable and integrated production strategies increasingly urgent [3]. Within this scenario, the Blue Economy has emerged as a strategic framework promoting sustainable marine resource use through the integration of economic development, environmental protection, and biodiversity conservation [4].

Among marine invertebrates, the Mediterranean Sea urchin *Paracentrotus lividus* (Lamarck,1816) represents a species of remarkable ecological and economic relevance. This echinoid is a keystone species for the regulation of macroalgal communities and the stability of marine food webs. At the same time, *P. lividus* gonads are a product with high commercial and gastronomic value, particularly in demand in Mediterranean and Asian markets. However, high fishing pressure, combined with the effects of climate change, habitat loss, and other anthropogenic pressures, has led to a significant reduction in natural populations in many areas of the Mediterranean, highlighting the need to develop innovative strategies for the sustainable management and conservation of the species [5].

In response to these challenges, sustainable aquaculture approaches have received growing attention. Integrated Multi-Trophic Aquaculture (IMTA) has emerged as a promising strategy capable of improving nutrient recycling, reducing environmental impacts, and increasing production efficiency through the integration of species occupying different trophic levels [6,7]. At the same time, advances in reproductive biotechnology and germplasm cryopreservation are opening new opportunities for the conservation of marine genetic resources and the development of more resilient aquaculture systems [8,9]. However, knowledge on *P. lividus* aquaculture, IMTA applications, sperm cryopreservation, and germplasm biobanking remains fragmented, as most studies have addressed these topics separately. An integrated framework linking sustainable production, biodiversity conservation, reproductive biotechnology, and genetic resource management is therefore still needed. Such an approach is particularly relevant for *P. lividus*, whose ecological role, commercial value, and increasing conservation concern make it a suitable model species for conservation-oriented aquaculture strategies. Although previous studies have investigated sperm cryopreservation in echinoids, standardized baseline data for sperm quality assessment and their application to germplasm banking remain limited in *P. lividus*. This gap limits the development of reproducible cryopreservation protocols and the practical implementation of germplasm biobanking strategies for conservation-oriented aquaculture.

Therefore, this narrative review, including a preliminary case study, aims to provide an integrated perspective on the role of *P. lividus* in sustainable Mediterranean aquaculture and marine biodiversity conservation. Specifically, the review discusses: (i) the ecological and economic relevance of the species; (ii) the main drivers contributing to the depletion of wild populations; (iii) current advances and constraints in echinoculture and IMTA systems including microbiological quality considerations; (iv) the importance of sperm quality assessment for reproductive management; and (v) the role of cryopreservation and germplasm biobanking as tools for genetic resource conservation.

Within this framework, the ongoing AVATIM research project (“Virtuous Aquaculture in Integrated Marine Multitrophic Aquaculture”), funded by the Italian Ministry of Agriculture, Food Sovereignty and Forestry, is presented as a preliminary operational case study aimed at developing integrated tools for sustainable multitrophic aquaculture and germplasm conservation. As a first step, baseline sperm quality parameters of sexually mature *P. lividus* males collected from the southern Adriatic Sea were assessed using Computer-Assisted Sperm Analysis and flow cytometry.

These preliminary data are intended to provide baseline values for future cryopreservation studies and germplasm biobanking strategies. In this perspective, AVATIM exemplifies how research on IMTA, reproductive biotechnology, and genetic resource conservation may contribute to a circular Blue Economy framework for the sustainable management of Mediterranean Sea urchin resources.

## 2. Literature Search Strategy and Review Methodology

The present narrative review was developed to critically analyse and integrate the available knowledge on *P. lividus* by connecting research areas that are generally addressed separately, including the conservation of natural populations, sustainable aquaculture, Integrated Multi-Trophic Aquaculture (IMTA) systems, sperm quality assessment, cryopreservation, and germplasm biobanking. Therefore, the aim of the review was not merely to provide a descriptive summary of the literature but rather to identify the main knowledge and methodological gaps, highlight unresolved and controversial issues, and propose an integrated perspective in which conservation aquaculture, reproductive technologies, and genetic resource management can contribute to the protection and sustainable use of the species.

The literature search and source-selection process were conducted according to a structured narrative approach, with the databases consulted, search-term combinations, and main criteria used to assess study relevance defined in advance. Given the interdisciplinary nature of the topic and the heterogeneity of the available studies, a quantitative meta-analysis was not performed. Instead, the selected publications were organised and interpreted through a thematic narrative synthesis.

### 2.1. Platforms and Databases Used for the Literature Search

The scientific literature was searched primarily using the Scopus and Web of Science Core Collection bibliographic databases, which were selected because of their broad coverage of international peer-reviewed publications in marine ecology, aquaculture, reproductive biology, and cryopreservation.

The searches were conducted in the main thematic fields covering titles, abstracts, and keywords. No initial restrictions were applied regarding publication year in order to include both fundamental historical studies and the most recent contributions. The search included publications available up to June 2026.

Google Scholar was used exclusively as a complementary tool to identify potentially relevant articles not retrieved through the main database searches, as well as institutional documents, book chapters, and publications cited in the selected studies. In addition, the reference lists of the most relevant articles and reviews were examined manually through backward citation searching to identify further relevant sources.

Information on fisheries and aquaculture production was supplemented, when necessary, with data from institutional sources and official databases, including those of the Food and Agriculture Organization of the United Nations.

### 2.2. Search Terms and Search Strategy

The search strategy was structured around four main thematic areas identified according to the objectives of the review. Specifically, the search addressed the ecology, population status, and conservation of *P. lividus*, as well as fisheries, aquaculture, and the potential integration of the species into Integrated Multi-Trophic Aquaculture systems. Additional areas of investigation included sperm cryopreservation and germplasm biobanking in aquatic organisms. A complementary search was also conducted on microbiological quality, host-associated microbiota, and their potential implications for animal health, food safety, and the biosafety of reproductive material.

For each thematic area, combinations of English-language terms were used together with the Boolean operators AND and OR. The full scientific name of the species was used to increase the specificity of searches focused on *P. lividus*, whereas broader terms were applied to aquatic biobanking because of the limited availability of studies specifically addressing sea urchins.

The following search strings were used:

- “*Paracentrotus lividus*” AND (ecology OR conservation);
- “*Paracentrotus lividus*” AND (fishery OR aquaculture OR IMTA);
- “*Paracentrotus lividus*” AND cryopreservation;
- (“germplasm banking” OR biobanking) AND aquatic;
- “*Paracentrotus lividus*” AND (microbiota OR microbiome OR “microbial community” OR “microbiological quality” OR pathogen* (The asterisk (*) was used as a truncation symbol to retrieve different word variants sharing the same root.) OR “food safety”).

The four main searches retrieved 158, 174, 16, and 7 records, respectively, in Scopus, and 1827, 740, 38, and 58 records, respectively, in Web of Science Core Collection. The complementary search on microbiological quality and host-associated microbiota retrieved 49 records in Scopus and 54 records in Web of Science Core Collection. The complete search strings and the corresponding numbers of retrieved records are summarised in Table 1.

The results obtained using the different search strings were not directly summed because the same publication could be retrieved by more than one search or indexed in both databases. The records were therefore assessed for thematic relevance and screened to remove overlapping entries. Additional targeted searches were conducted during manuscript preparation whenever specific topics required further investigation, including sperm quality, CASA systems, the physiology of sperm activation, cryoprotectants, cooling and warming rates, fertilization, larval development, and the conservation of genetic diversity.

### 2.3. Inclusion and Exclusion Criteria

During title and abstract screening, records that were not directly relevant to the ecology and conservation of *P. lividus*, echinoculture and IMTA systems, sperm quality, cryopreservation, germplasm biobanking, microbiological quality, or host-associated microbiota were excluded. Duplicate records, conference abstracts, editorial letters, and contributions for which the full text was unavailable were also excluded.

During full-text assessment, studies that did not provide sufficient biological, methodological, or applied information in relation to the objectives of the review were excluded. Studies conducted on other aquatic species were retained only when they provided methodological or conceptual information directly applicable to cryopreservation, biobanking, sperm physiology, or IMTA systems involving echinoderms.

General literature concerning ecology, reproductive cycles, population status, and environmental pressures was retained as contextual evidence. Studies on cryopreservation were assessed with particular attention to the availability of information on extender composition, cryoprotectant type and concentration, freezing and thawing procedures, and sperm quality parameters.

### 2.4. Data Extraction and Synthesis

Because the included studies differed substantially in terms of species, objectives, experimental design, and methodologies, a meta-analysis was not considered appropriate. The available evidence was therefore integrated through a thematic narrative synthesis.

For cryopreservation studies, the information extracted, when available, included species, semen collection method, extender composition, cryoprotectant type and concentration, equilibration time, packaging format, cooling rate, thawing procedure, and post-thaw parameters, including motility, viability, and fertilising capacity. For the other thematic areas, the main information extracted concerned the ecological role of *P. lividus*, pressures affecting natural populations, limitations of echinoculture, applications in IMTA systems, and the future potential of germplasm biobanking.

For microbiological studies, the information considered included microbial communities associated with different host compartments, the presence of potentially pathogenic microorganisms, product safety, and possible implications for the biosafety of aquaculture systems, hatcheries, and reproductive material.

Finally, the literature was organised into thematic sections, with particular attention given to knowledge gaps, methodological differences among studies, unresolved issues, and priorities for future research.

## 3. The Sea Urchin, *Paracentrotus lividus*, as a Key Species in the Mediterranean Ecosystem

Sea urchins (Class Echinoidea, Phylum Echinodermata) are benthic invertebrates widely distributed in marine ecosystems, where they play a fundamental role in structuring benthic communities [10,11]. Among the different species, *P. lividus* is one of the most common echinoids in the Mediterranean Sea and northeastern Atlantic, with a distribution ranging from the British Isles to North Africa and several Atlantic archipelagos [12].

Through their grazing activity, sea urchins act as key ecosystem engineers by controlling macroalgal biomass, influencing benthic community composition, and contributing to nutrient cycling [13,14]. Their feeding behavior, enabled by Aristotle’s lantern, allows efficient exploitation of hard substrates and a wide range of benthic resources, including macroalgae, epiphytic microalgae, microbial biofilms, and organic material [15]. In this way, sea urchins contribute to trophic processes, ecological succession, and habitat availability for associated benthic species.

At moderate densities, *P. lividus* contributes to maintaining the balance between algal communities and benthic fauna, whereas fluctuations in its abundance can substantially alter ecosystem structure [16]. In particular, one of the ecological phenomena most strongly associated with sea urchin populations is the formation of so-called barren grounds, habitats characterized by rocky substrates dominated by encrusting coralline algae, sparse macroalgal cover, low productivity, and reduced biodiversity [17,18,19]. The ecological role of sea urchins is strongly modulated by predation pressure, habitat complexity, and trophic context. Predators such as gastropods (*Hexaplex trunculus*), starfish (*Marthasterias glacialis*), and benthic fish assemblages can regulate sea urchin abundance and behavior, particularly in structurally complex habitats such as *Posidonia oceanica* meadows, which may act as refuges or foraging areas depending on predator presence and local habitat conditions [20,21]. Structurally complex seagrass meadows can provide protection from predators [22], although predation risk may vary depending on habitat type, benthic predator abundance, and broader food-web interactions [23,24]. In addition, predatory fish communities, particularly in marine protected areas, can enhance predation pressure and influence sea urchin distribution, sometimes driving individuals toward seagrass refuges [25].

When sea urchin populations exceed critical density thresholds, grazing pressure can become unsustainable, leading to overgrazing and the development of urchin barrens. The transition from macroalgal forests to barren grounds represents a classic regime shift, often driven by trophic imbalance, reinforced by predator loss, and stabilized by positive feedback mechanisms [13]. Conversely, regulated harvesting and targeted removal of sea urchins can promote the recovery of algal forests by reducing excessive grazing pressure [26,27].

Overall, the ecological role of *P. lividus* is density-dependent and context-specific. While balanced populations support benthic ecosystem functioning, excessive or poorly regulated densities may contribute to habitat degradation. This dual role highlights the importance of integrating population management, habitat conservation, and sustainable aquaculture strategies for the long-term conservation and use of this species.

## 4. Decline of Natural Populations and Environmental Pressures

The decline of *P. lividus* populations observed in numerous areas of the Mediterranean cannot be attributed to a single cause but rather results from the interaction of multiple anthropogenic and environmental pressures. Overexploitation, climate change, habitat degradation, and contamination act at different spatial and temporal scales, influencing the species’ survival, growth, reproductive success, and recruitment. Understanding these pressures is essential to identify effective strategies for conservation and sustainable development of echinoculture.

The gonads of *P. lividus* are particularly prized by consumers and have become one of the most sought-after seafood delicacies worldwide. Market demand has increased substantially over recent decades, with retail prices frequently exceeding €150 kg^−1^ depending on product quality, season, and market destination [28]. This growing demand, combined with the limited availability of natural stocks, has contributed to increasing fishing pressure on wild populations and has stimulated interest in the development of sustainable aquaculture production systems.

### 4.1. Overexploitation and Fishing Pressure

Beyond its ecological importance, *P. lividus* represents a marine resource of considerable economic value throughout the Mediterranean region. According to FAO statistics, the global natural harvest of sea urchins ranged from a peak of 42,978 tonnes in 2014 to 30,815 tonnes in 2023, while aquaculture production, after reaching 16,205 tonnes in 2021, declined to 8150 tonnes in 2023 [29].

In the Mediterranean countries such as Italy, France, Spain, and Greece, sea urchin fisheries significantly contribute to local economies and support numerous small-scale artisanal fisheries [28,30]. Several sea urchin species are commercialized for their edible gonads, known as “roe”, “caviar” or “uni”, which are highly appreciated in international markets due to their distinctive organoleptic characteristics, nutritional value, and quality color [31].

Unfortunately, in areas with high demand, *P. lividus* populations are exposed to multiple pressures that affect their distribution, demographic structure, and reproductive capacity. These issues, combined with seasonal availability, contributed to unsustainable harvesting regimes, often associated with illegal or poorly regulated fishing practices.

Among anthropogenic factors, commercial fishing for human consumption represents a major source of impact [32,33]. Echinoderms are reported as a category of seafood illegally caught worldwide [1,34]. Its environmental, economic, and social consequences make it an international problem also related to sanitary risks with potentially serious implications for the health of the final consumer.

Over recent decades, such exploitation pressure has been linked to marked declines in natural populations, with harvest rates frequently exceeding the species’ capacity for demographic recovery [5]. In addition, population fragmentation and reduced connectivity among local stocks further constrain recovery potential, limiting larval replenishment and increasing overall vulnerability to environmental variability and other stressors.

### 4.2. Climate Change and Environmental Stressors

In addition to direct pressures related to exploitation, *P. lividus* populations are regulated by a complex set of environmental factors acting at multiple spatial scales. At broad scales, variables such as temperature and primary production influence larval dispersal processes and species distribution [35]. At intermediate and smaller scales, local factors play a key role, including wave exposure, trophic availability, habitat characteristics, and the intensity of anthropogenic activities [36,37,38,39].

Moreover, warming events have been associated with mass mortality events and range contractions, particularly in the warmer regions of the Mediterranean, where the exceedance of critical thermal thresholds (~30–31 °C) significantly increases adult mortality [35]. Environmental variability also affects reproductive cycles, potentially reducing synchrony in gamete release [40].

In parallel, climate change is emerging as a key driver in the regulation of echinoid communities. Increasing seawater temperatures are leading to shifts in community structure and functional composition, favoring the expansion of thermophilic species and altering trophic interactions [41,42]. In the Mediterranean Sea, the growing predominance of *Arbacia lixula* (black sea urchin) over *P. lividus* represents a clear example of benthic community reorganization associated with warmer conditions. Indeed, *A. lixula* densities have increased significantly over recent decades [43], indicating a potential response to changing environmental conditions.

Although both species are herbivorous, they differ substantially in their grazing modes and impacts on habitat structure. *A. lixula* exhibits a greater ability to scrape rocky surfaces [12] and is subject to lower predation pressure than *P. lividus*, likely due to specific morphological traits such as stronger attachment to the substrate, a more robust test, and longer spines [44,45]. Consequently, an increase in its abundance, or in the *A. lixula*: *P. lividus* ratio, can lead to higher grazing intensity on rocky substrates, with significant effects on algal cover and benthic habitat structure [46]. Unlike *P. lividus*, however, *A. lixula* is generally not targeted by Mediterranean fisheries for human consumption because its gonads are smaller and its organoleptic characteristics are less appreciated, resulting in little commercial value [33].

### 4.3. Pollution and Emerging Contaminants

Another threat to *P. lividus* populations is the contamination of coastal environments, which is particularly severe in areas subjected to strong anthropogenic pressure. Marine sediments act as important reservoirs of pollutants, including heavy metals, polycyclic aromatic hydrocarbons (PAHs), and other persistent compounds, which can be remobilized and become bioavailable to benthic organisms [47]. In this context, *P. lividus* can bioaccumulate a broad range of persistent and emerging contaminants in its gonadal tissues, although the concentrations reported in wild specimens were generally low [48].

A growing body of experimental evidence indicates that exposure to environmental pollutants can induce developmental, morphological, and molecular alterations during the early life stages of *P. lividus*, confirming the high sensitivity of embryos and larvae to chemical stressors [47,49].

In recent years, particular attention has been directed towards microplastics, which are widespread contaminants of marine ecosystems. Experimental and meta-analytical evidence indicates that microplastic exposure can negatively affect fundamental biological processes in marine benthic organisms, including development, feeding, growth, survival, and reproduction [50]. In *P. lividus*, in vitro exposure of spermatozoa to polystyrene microplastics reduced cell viability and motility and induced reactive oxygen species-mediated DNA fragmentation, indicating potential adverse effects on reproductive function [51]. In addition, chemicals released from or associated with plastic particles may contribute to their overall toxicity [50].

Recent evidence has also shown that the toxicity of emerging contaminants may be modulated by climate-related environmental conditions. In *P. lividus*, combined exposure to the organic UV filter octinoxate and elevated temperature induced marked oxidative stress, metabolic alterations, and changes in detoxification responses. Conversely, increased salinity partially attenuated these effects, although biochemical alterations remained detectable [52].

Finally, simultaneous exposure to chemical pollutants and climate-related stressors, including ocean warming and acidification, can produce complex additive, synergistic, or antagonistic responses. Experimental studies on *P. lividus* have shown that combined exposure to microplastics, reduced pH, and elevated temperature may impair larval growth and development, potentially increasing the vulnerability of early life stages under future environmental conditions [53].

Beyond reducing adult abundance, environmental pressures may also affect reproductive performance and recruitment success. Thermal stress, pollution, and emerging contaminants can interfere with gamete quality, fertilization processes, embryonic development, and larval survival, thereby reducing the resilience of natural populations [35,40,47,49,54]. In this context, the availability of genetic material preserved in biobanks may represent a precautionary and strategic resource to preserve genetic variability, support the selection of genotypes of interest, and assist future conservation-oriented aquaculture strategies for depleted or vulnerable *P. lividus* populations [55,56].

## 5. Sustainable Aquaculture of *Paracentrotus lividus*

### 5.1. State of the Art of Echinoculture

Over the last two decades, significant advances have been achieved in broodstock conditioning, larval culture, juvenile production, and roe enhancement techniques [57,58]. In recent years, knowledge of the biological cycle of the sea urchin has been useful in aquatic ecotoxicity tests [59,60].

Current production approaches include both full life-cycle culture and gonad enhancement of wild-collected individuals through controlled feeding regimes. Despite these improvements, the sector remains relatively small compared with other aquaculture industries, mainly due to biological constraints affecting production efficiency and economic sustainability. In particular, larval rearing, juvenile availability, and feed optimization continue to represent major challenges for large-scale commercialization.

Despite increasing research and technological development, *P. lividus* echinoculture has not yet reached the level of a consolidated large-scale aquaculture industry. Most reported Mediterranean and European initiatives remain experimental, pilot-scale, or pre-commercial, and species-specific production statistics are still limited. Several production models have been investigated, including land-based flow-through and recirculating aquaculture systems for broodstock conditioning, larval and juvenile production, and gonad enhancement, as well as open-water and sea-based culture systems [57,61,62]. Land-based systems provide greater control over water quality, reproduction, feeding, and biosecurity but require higher infrastructure, water treatment, and energy inputs, whereas sea-based systems may reduce some operating costs but provide less control over environmental variability, predation, and biofouling. Overall, reliable juvenile production, larval survival, relatively slow growth, feed optimization, gonad-quality standardization, and production costs remain major barriers to large-scale commercialization [63,64].

### 5.2. Main Production Constraints

The industrial development of *P. lividus* aquaculture is still constrained by several biological and technological bottlenecks. Among these, slow growth rates, limited availability of hatchery-produced juveniles, high larval sensitivity to environmental conditions, and the lack of optimized feeding protocols remain the most significant challenges [61,62].

The time required to reach commercial size represents an additional constraint for the development of *P. lividus* aquaculture. A minimum commercial size of approximately 50 mm test diameter (excluding spines) has been reported, which may require approximately 3–3.5 years under farming conditions [5]. However, growth rates and size-at-age can vary considerably depending on temperature, food availability, and local environmental conditions. Harvesting requirements may also differ among countries and regions; in Italy, for example, the minimum harvestable size is set at 7 cm in diameter, including spines [5].

Larval production represents a critical phase of the culture cycle. Early developmental stages are highly sensitive to water quality, microbial dynamics, and nutritional conditions, often resulting in elevated mortality rates and increased production costs [63,64]. In addition, the continued reliance on wild broodstock limits the possibility of fully closing the life cycle under captive conditions and may contribute to further pressure on natural populations.

Nutrition is another key factor influencing both production performance and market value. Gonad quality, the principal commercial trait, is strongly affected by diet composition, influencing gonad size, pigmentation, texture, and nutritional characteristics [65,66,67]. Although formulated feeds and alternative ingredients have shown promising results, no universally optimized diet currently exists that simultaneously maximizes growth, gonad quality, and economic sustainability [68,69].

Overall, overcoming these constraints will require the integration of advances in hatchery technology, larval rearing, nutrition, and reproductive biotechnology to improve the efficiency and sustainability of Mediterranean echinoculture. The principal limitations currently hindering the expansion of *P. lividus* aquaculture, together with their implications and future research priorities, are summarized in Table 2.

### 5.3. Genetic Selection and Resilience

A further avenue for improving echinoculture concerns the application of genetic selection programs, which are already well established in many fish and shellfish species but are still in the early stages of development for sea urchins. Such approaches could represent a complementary strategy to innovations in nutrition and technology, contributing to improved production efficiency and reduced dependence on natural populations.

Key traits of interest include growth rate, gonad yield, product quality, and resistance to environmental stressors. In particular, rising sea temperatures and the increasing frequency of extreme climatic events are making thermal tolerance an extremely relevant trait for both aquaculture and the conservation of natural populations.

The integration of genomic tools and breeding programs could enable the identification of genetically superior and more resilient lines, thereby improving production efficiency and adaptive capacity under future climate scenarios.

Quantitative evidence in species of sea urchins such as *Strongylocentrotus intermedius* has shown that commercially relevant traits, including body weight, test diameter, and gonad quality, exhibit moderate to high heritability, indicating significant potential for improvement through selective breeding [70,71]. Similarly, studies on families of sea urchins exposed to different thermal conditions have revealed genetic variability in stress responses, suggesting the possibility of selecting lines with greater resilience to heatwaves [72].

Despite these opportunities, in the Mediterranean context and for *P. lividus*, the implementation of structured genetic strategies is still limited. Studies on experimental restocking programs have shown that the absence of advanced genetic management can reduce genetic diversity and increase inbreeding in cultured populations. Moreover, molecular analyses have demonstrated that maternal effects and rearing conditions influence fertilization success, larval development, and the expression of genes related to stress response and skeletogenesis, highlighting the importance of optimizing rearing protocols for the sustainable production of the species [54,55].

In this context, the availability of genetic material preserved in biobanks could represent a strategic resource for managing genetic variability and selecting genotypes of interest [56].

### 5.4. Paracentrotus lividus in IMTA Systems

IMTA is considered one of the most promising strategies for improving environmental sustainability and the efficiency of aquaculture production systems. Unlike conventional systems based on monoculture, IMTA integrates organisms belonging to different trophic levels, enabling the recovery and reuse of nutrients and organic matter derived from production activities. This approach promotes resource recycling, reduces the environmental impacts associated with intensive aquaculture, and supports production models that are more consistent with the principles of the circular economy and the Blue Economy [6,7].

In this context, sea urchins are gaining increasing interest as functional components of IMTA systems due to their ability to utilize macroalgal biomass, biofilms, and organic material derived from aquaculture activities. Among Mediterranean species, *P. lividus* represents one of the most promising candidates for integration into multitrophic systems due to its high ecological plasticity, broad dietary spectrum, and the high commercial value of its gonads [57,69].

Early experimental evidence has demonstrated that *P. lividus* can be effectively integrated into systems associated with fish farming and macroalgae cultivation. In 2018, Shpigel et al. [69] developed an IMTA system comprising gilthead seabream, *Ulva lactuca*, and *P. lividus*, demonstrating the sea urchin’s ability to efficiently convert nutrients from other trophic compartments into gonadal biomass. Subsequently, Grosso et al. [73] proposed an innovative multitrophic system in which *P. lividus* was the main species and the sea cucumber *Holothuria tubulosa* acted as an extractive species, highlighting how the integration of different trophic compartments can enhance the reuse of organic matter and reduce the accumulation of organic waste.

More recently, Wang et al. [74] compared monoculture systems with shrimp–sea urchin IMTA systems, observing improvements in water quality and significant differences in sediment microbial communities, confirming the potential of IMTA to promote nutrient recycling processes and greater ecological stability. Similarly, Santos et al. [75] evaluated the integration of sea urchins and polychaetes in a system aimed at valorising aquaculture effluents, demonstrating that the association between herbivorous and detritivorous organisms can increase the utilization of residual organic matter and improve overall system efficiency.

Beyond the production of high-value biomass, the integration of *P. lividus* into IMTA systems can provide important ecosystem services. Through grazing on macroalgal communities and organic deposits, sea urchins can contribute to biofouling control, enhance nutrient recycling, and limit the accumulation of organic matter near farming facilities. These functions may help mitigate some of the environmental impacts commonly associated with intensive aquaculture.

Despite these promising prospects, the large-scale application of IMTA systems based on *P. lividus* is still limited by several biological, technological, and economic factors. The relatively slow growth rate of the species, the not always consistent availability of hatchery-produced juveniles, and the need to develop sustainable and cost-effective diets remain important constraints for the development of echinoculture [12,63]. Further studies are therefore needed to optimize species combinations, quantify environmental and economic benefits, and assess the sustainability of such systems at a commercial scale.

Overall, the integration of *P. lividus* into IMTA systems represents a promising strategy for combining sustainable aquaculture production, nutrient recovery, circular economy principles, and marine biodiversity conservation. In this perspective, the sea urchin should not be considered solely a commercial species but also a functional component of more resilient, efficient, and ecologically sustainable aquaculture systems. However, the development of *P. lividus*-based IMTA systems requires a reliable supply of juveniles for stocking and production purposes. Repeated reliance on wild broodstock for hatchery production would be inconsistent with the conservation-oriented rationale of IMTA, especially where natural populations are already depleted or vulnerable [5]. Moreover, hatchery production based on a limited number of breeders may increase the risk of reduced genetic diversity and inbreeding, highlighting the need for appropriate genetic management [55].

Within conservation-oriented aquaculture, IMTA, hatchery production, and germplasm biobanking should therefore be considered complementary rather than intrinsically interdependent tools. IMTA primarily addresses production efficiency and nutrient recycling, whereas hatcheries provide the juveniles required for stocking and production. Sperm biobanking may complement hatchery management by preserving paternal genetic resources from multiple donors and allowing their use across reproductive seasons. However, sperm cryopreservation does not replace the need for female broodstock, viable oocytes, appropriate fertilization procedures, or hatchery infrastructure.

### 5.5. Microbiological Quality and Host-Associated Microbiota

In sustainable echinoculture and IMTA, microbiological quality and host-associated microbial communities represent two related but distinct emerging aspects for the management of *P. lividus*. Recent microbiome studies have shown that sea urchins can be considered holobionts, with microbial communities varying according to species, body compartment, and geographic location. In *P. lividus*, coelomic fluid, coelomocytes, and fecal pellets host distinct microbial assemblages that may be involved in host–microbe interactions, digestion, and nutrient cycling [76,77].

By contrast, microbiological safety monitoring is directed towards the detection or quantification of microorganisms that may affect animal health, production systems, or consumer safety. This distinction is particularly relevant from a production and consumer safety perspective because *P. lividus* gonads are a high-value edible product that may be consumed raw or minimally processed. The assessment of water, biofilms, gonads, and coelomic fluid could therefore contribute to the development of microbiological surveillance and risk-management strategies for future *P. lividus* aquaculture systems, including open-sea IMTA models [78]. Disease-associated bacteria have been linked to pathological conditions in *P. lividus*, including bald sea urchin disease [79]. Evidence from other edible sea urchins indicates that diet may modulate intestinal microbiota and roe quality, suggesting a possible contribution of microbiome-related indicators to the assessment of animal health and product quality in echinoculture [80].

The possible relevance of microbiological monitoring to *P. lividus* hatchery and germplasm biobanking workflows remains largely unexplored. Microbial contamination of water, biofilms, coelomic fluid, or reproductive material could potentially affect sample quality or introduce undesirable microorganisms during gamete collection, processing, and cryostorage. However, specific studies on the microbiological quality of *P. lividus* semen and cryopreserved germplasm are currently lacking, and the potential role of microbiological monitoring in *P. lividus* germplasm biobanking remains to be investigated. At present, this rationale is supported only indirectly by studies on *P. lividus* rearing systems and by evidence from cryopreserved semen or biobanking practices in other animal models [78,81].

Dedicated studies are therefore needed to determine whether microbial contamination affects the quality, post-thaw performance, or biosafety of cryopreserved *P. lividus* germplasm and to establish whether species-specific monitoring procedures are warranted.

Overall, microbiome characterization may improve understanding of host–microbe interactions and their relationships with nutrition, health, and environmental conditions, whereas microbiological safety monitoring may support the management of animal health and edible-product safety [76,77,78,79,80]. Further research is needed to define the potential role and appropriate scope of microbiological monitoring within *P. lividus* hatchery production and germplasm biobanking [78,81].

## 6. Cryopreservation and Germplasm Biobanks

### 6.1. Cryopreservation in Aquatic Organisms

Cryopreservation represents one of the most valuable reproductive biotechnologies for the management of aquatic genetic resources, supporting aquaculture development, selective breeding programs, biodiversity conservation, and restocking initiatives. In marine environments, sperm cryobanking can support genetic management and provide preserved paternal genetic resources for conservation and appropriately designed restocking programs. However, sperm cryopreservation cannot independently enable population recovery, which also depends on female gamete availability, hatchery production, habitat suitability, appropriate genetic management, and in situ conservation measures [82].

Research on cryopreservation in aquatic organisms has primarily focused on spermatozoa [9,83], although oocytes, embryos, and primordial germ cells have also been investigated [84,85,86,87]. Among these, spermatozoa are currently the most suitable for cryobanking due to their small size, low cytoplasmic content, and relatively higher resistance to osmotic and thermal stress. In contrast, oocytes and embryos remain more difficult to preserve because of their larger size, high water content, and susceptibility to intracellular ice formation [83,88].

The two main cryopreservation approaches are controlled-rate freezing and vitrification. Both aim to minimize cellular damage caused by ice crystal formation, osmotic imbalance, membrane disruption, and oxidative stress, which can compromise post-thaw viability and fertilization capacity [89,90,91]. The success of a protocol depends on the preservation of both structural and functional cell integrity.

Several factors influence cryopreservation outcomes, including cryoprotectant type and concentration, cooling and warming rates, storage conditions, and species-specific biological traits [9,92]. Permeating cryoprotectants such as dimethyl sulfoxide (DMSO), ethylene glycol, glycerol, and methanol reduce intracellular ice formation, whereas non-permeating agents such as sucrose and polyvinylpyrrolidone mitigate osmotic stress [93].

Beyond technical considerations, cryopreservation provides key advantages for aquaculture and conservation. Cryobanked genetic resources can be stored independently of reproductive seasonality, supporting selective breeding and restoration programs, while potentially reducing reliance on wild broodstock [94]. In addition, standardized cryopreserved material improves reproducibility in ecotoxicological studies and ensures year-round availability of biological samples for research and production systems [95].

### 6.2. Paracentrotus lividus: Current Status and Challenges in Cryopreservation

Despite advances in aquatic cryobiology, sea urchin cryopreservation remains largely restricted to spermatozoa, while oocytes and embryos are still poorly investigated. Sperm cryopreservation is currently the most promising approach for germplasm conservation in echinoids; however, its application is limited by methodological variability and poor protocol standardization.

In *P. lividus*, cryopreservation success depends on multiple interacting factors, including sample preparation, extender composition, cryoprotectant selection, sperm concentration, equilibration time, and freezing–thawing conditions. Table 3 shows some of the main biological and technical factors that influence sperm cryopreservation in *P. lividus*, based on the literature.

Sample preparation is a critical step. Extenders, typically based on saline solutions or seawater [96,97], influence post-thaw sperm quality by reducing enzymatic activity in seminal plasma [98] and protecting cells from oxidative stress and ice-induced damage [99]. This is particularly relevant in sea urchins, where reactive oxygen species (ROS) production increases after motility activation and is tightly linked to cellular respiration, directly affecting sperm functionality.

Cryoprotectant selection is another key factor and is highly species-specific [100]. In echinoids, only a limited number of species have been studied, and transferable protocols remain scarce [101]. Among permeating cryoprotectants, DMSO and methanol are widely used and often outperform glycerol, as reported also in other marine invertebrates such as *Haliotis diversicolor* [100] and in sea urchin species such as *Hemicentrotus pulcherrimus* and *Strongylocentrotus intermedius*.

Additives such as trehalose can further improve cryoprotection by stabilizing cellular membranes during freezing and thawing [102,103].

Among available protocols for *P. lividus*, the best results have been obtained using 7% DMSO in 1% NaCl supplemented with 0.04 M trehalose [104]. The small size of sperm cells facilitates rapid cryoprotectant diffusion, reducing toxicity risks [97].

A major unresolved issue is the lack of systematic studies on sperm concentration and dilution ratios. This represents a critical limitation for protocol scalability, as sperm density strongly influences post-thaw motility and fertilization performance in several species [105,106,107].

Equilibration time is another species-dependent parameter. In sea urchins, short equilibration periods (around 10 min at 4–18 °C) are generally effective for cryoprotectant penetration [104], although interspecific variability suggests that this parameter requires further optimization.

Cooling rate is a key determinant of cryopreservation success. In aquatic species, rates between 5 and 100 °C min^−1^ have been reported [97,100,108]. For *P. lividus*, intermediate cooling rates, approximately 20 °C min^−1^, appear to provide the best results, achieving a balance between intracellular ice formation and excessive cellular dehydration. At the same time, simpler and more cost-effective cooling systems, such as rate-controlled passive devices, are attracting growing interest as viable alternatives to programmable freezers [109].

Considering where to freeze the sample, although straws generally ensure better thermal uniformity than cryovials [110], some studies report improved outcomes using 2 mL tubes, indicating that container type is also a relevant optimization factor.

Overall, most studies focus on immediate post-thaw endpoints such as motility, viability, and fertilization rate, while long-term effects on larval development, offspring fitness, and transgenerational stability remain poorly understood.

While cryopreservation in sea urchins has progressed from proof-of-concept to an applicable technology, its translation into routine biobanking is still limited by methodological variability and lack of standardization.

**Table 3:** Main biological and technical factors influencing sperm cryopreservation success in *Paracentrotus lividus*.

Factor	Main Role in Cryopreservation	Effect on Post-Thaw Quality	Notes/Optimal Range (If Available)	Reference
Extender composition	Osmotic balance, enzymatic inhibition, ROS protection	Influences sperm survival and functionality	Saline solutions or seawater-based extenders	[96,97,98,99]
Cryoprotectant type	Prevents intracellular ice formation	Balances protection vs. toxicity	DMSO and methanol are the most effective	[96,100,104]
Cryoprotectant concentration	Controls toxicity and permeability	High doses increase toxicity; low doses reduce protection	e.g., 7% DMSO commonly used	[97,104]
Dilution ratio	Sperm-CPA-extender balance	Influences final sperm density and survival	Highly variable among protocols	[104]
Equilibration time	CPA penetration into cells	Too short: insufficient protection; too long: toxicity	~10 min commonly used (4–18 °C)	[104]
Cooling rate	Ice crystal formation vs. dehydration	Slow: dehydration; fast: intracellular ice	Optimal ~20 °C min^−1^	[104]
Container type	Thermal conductivity and uniformity	Affects freezing consistency	Straws vs. 2 mL tubes	[104]
Warming rate	Recrystallization control	Rapid thawing generally improves survival	Often underestimated parameter	[104,109]

### 6.3. Germplasm Biobanks

Germplasm biobanks are infrastructures dedicated to the collection, characterization, cryopreservation, and long-term storage of genetic material, including spermatozoa, oocytes, embryos, and germ cells [111,112]. Although biobanking practices were initially consolidated in biomedical research and livestock breeding, they are increasingly being integrated into wildlife conservation programs to safeguard genetic diversity and support reproductive research, conservation breeding, and population management [112,113].

In marine systems, germplasm biobanks provide a strategy for preserving genetic diversity in species exposed to anthropogenic pressure, such as *P. lividus*. *Ex situ* preservation allows the maintenance of genetic material from different populations, in this way helping to limit genetic erosion and providing genetic resources that may support appropriately designed future restoration or restocking programs.

Biobanks may also preserve genetically characterized material of potential interest under environmental change and could, in the future, contribute to assisted-evolution approaches aimed at enhancing resilience to stressors such as warming and acidification.

In addition to their role in conservation, germplasm biobanks can support aquaculture production and breeding programs by synchronizing gamete availability, simplifying broodstock management, facilitating the transport of gametes among facilities, and preserving genetic material for selective breeding [114,115]. More broadly, controlled reproduction in cultured fish can be enhanced through hormonal management of sperm production and quality [116].

However, their implementation in sea urchins is still constrained by the lack of standardized protocols, high costs, and limited knowledge of long-term biological effects. For this reason, biobanks should be considered complementary tools to in situ conservation rather than replacements [117].

#### Challenges for Cryobanking in Sea Urchins

Current evidence indicates that cryopreservation is a feasible approach for preserving sea urchin genetic resources. Beyond simple storage of gametes, cryobanks can support hatchery systems by ensuring long-term access to genetically characterized material, reducing dependence on wild broodstock. This is particularly relevant for *P. lividus*, whose production still relies heavily on seasonal wild collection.

Standardized cryopreserved material can also support research in developmental biology, ecotoxicology, and climate change, improving reproducibility and reducing sampling pressure on natural populations.

Despite these advantages, several limitations persist. Cryopreservation induces cellular stress affecting membranes, nuclei, cytoplasm, and flagella, leading to DNA damage, reduced motility, and lower fertilization success [115,118]. Species-specific variability in gamete physiology and cryoprotectant sensitivity further complicates protocol standardization.

Moreover, little is known about long-term consequences on offspring performance and genetic stability across generations. Effective biobanking also requires robust sampling strategies to ensure that stored material accurately reflects population genetic diversity [119].

In addition, standardized procedures for sample collection, aseptic handling, donor screening, and microbiological quality control should be considered as complementary requirements to ensure the biological safety, traceability, and reproducibility of cryobanked material intended for aquaculture, hatchery production, or conservation applications [78,81]. Future research should therefore integrate cryopreservation optimization with evaluations of reproductive success, larval development, post-thaw sperm functionality, microbiological safety, and long-term genetic integrity, with the aim of transforming cryobanking into a fully operational conservation tool.

### 6.4. From Germplasm Biobanks to Conservation Aquaculture

Traditionally, germplasm biobanks have been viewed as passive repositories of genetic resources. More recently, they have increasingly been regarded as complementary components of conservation aquaculture systems, in which cryopreserved material can facilitate selective breeding, genetic management, and appropriately designed restocking programs.

Within this framework, biobanks should be integrated into broader conservation strategies rather than operating as isolated infrastructures. Cryopreserved germplasm may contribute to the development of resilient aquaculture stocks and the maintenance of genetic diversity and may provide preserved genetic resources for broader population-recovery programs when integrated with hatchery production, appropriate genetic management, and in situ conservation measures.

This integrated perspective positions cryobanking as a key enabling technology for a sustainable Blue Economy, linking biodiversity conservation with aquaculture innovation and marine resource management.

### 6.5. An Integrated Sustainability Framework: The AVATIM Project

Future Mediterranean aquaculture will increasingly require strategies that combine production efficiency with biodiversity conservation and genetic-resource management. Within this framework, conservation-oriented aquaculture may complement conventional production by integrating hatchery technologies and *ex situ* genetic-resource preservation with environmentally sustainable farming approaches.

*P. lividus* represents a relevant model for exploring this integrated perspective because of its ecological importance, commercial value, and potential inclusion in IMTA systems. The ongoing AVATIM project (“Virtuous Aquaculture in Integrated Marine Multitrophic Aquaculture”) was conceived to investigate this framework by combining offshore IMTA, reproductive biotechnology, and germplasm conservation. Within the project, IMTA is intended to address production diversification, resource-use efficiency, and nutrient recovery, whereas sperm cryobanking is being investigated as a complementary tool for preserving and managing paternal genetic resources.

As AVATIM is an ongoing project, this integrated framework represents the project’s intended objectives and research strategy rather than a set of fully validated outcomes. At present, the empirical results available from the project are limited to the preliminary characterization of fresh sperm quality described in Section 7. The performance of cryopreserved sperm and the broader effectiveness of the proposed integrated framework remain to be experimentally evaluated.

## 7. Preliminary AVATIM Case Study: Baseline Sperm Quality Assessment for Future Germplasm Biobanking

### 7.1. Case-Study Framework and Sperm Quality Assessment

A fundamental step toward the establishment of a germplasm biobank is the characterization of reproductive traits and sperm quality parameters under controlled conditions.

For this reason, the initial activities carried out within the AVATIM project focused on the standardization of sperm collection procedures and the establishment of baseline reproductive and sperm quality parameters for *Paracentrotus lividus* males. Particular attention was devoted to sperm concentration, viability, and motility characteristics, as these variables represent key indicators of gamete quality and are commonly used to evaluate cryopreservation efficiency and determine sample suitability for long-term germplasm conservation [104].

To achieve these objectives, adult specimens of *P. lividus* were collected along the southern Adriatic coast of Italy from an artificial rocky ledge near Bari, Apulia, Italy (40°59′31″ N, 17°13′17″ E) during March–May 2026. A total of 70 adult *P. lividus* specimens were considered for gamete collection. Following KCl-induced spawning, semen was successfully obtained from 35 sexually mature males, which were included in the sperm-quality assessment. The analyzed males had a mean body weight of 33.84 ± 5.63 g and a mean test diameter of 7.17 ± 0.30 cm, corresponding to the size range associated with sexual maturity, following the criteria reported by Zupo et al. [120] and Miroglio et al. [60].

Gamete release was induced by intracoelomic injection of 1 mL of 0.5 M KCl. Sperm was collected individually using a dry collection method to minimize premature activation and avoid sample contamination prior to analysis.

Sperm quality was assessed immediately after collection using standardized procedures commonly applied in marine invertebrate reproductive biology. The evaluated parameters included ejaculate volume, sperm concentration, sperm motility, and sperm membrane integrity.

Ejaculate volume was determined immediately after sperm collection by measuring the total volume of semen released by each individual using a calibrated micropipette. Values were recorded individually and expressed in milliliters (mL).

Sperm concentration was determined using a Neubauer haemocytometer. Semen samples were diluted at a ratio of 1:1000 (*v*/*v*) with 3% (*w*/*v*) NaCl solution, and spermatozoa were counted in duplicate under 400× magnification. Results were expressed as ×10^9^ spermatozoa mL^−1^.

Fresh semen was analysed immediately after collection. Sperm motility was evaluated following activation in filtered seawater at a salinity of 35‰ [121] and analysed using a Computer-Assisted Sperm Analysis (CASA) system operated through Sperm Class Analyser^®^ software (SCA^®^, version 6.3.0.59, Microptic S.L., Barcelona, Spain). Following activation, CASA video acquisition was initiated immediately. Sperm movement was recorded using a high-speed digital camera (Basler A602f-2; resolution: 782 × 582 pixels; 100 frames s^−1^) mounted on a Nikon Eclipse E600 phase-contrast microscope (Nikon Instruments, Florence, Italy), equipped with a total magnification of 100×. The SCA^®^ acquisition parameters were set as follows: minimum particle area, 50 µm^2^; maximum particle area, 400 µm^2^; frame rate, 100 frames s^−1^; and 100 consecutive images per acquisition. Three replicate recordings were analysed for each sample, with approximately 250–500 sperm tracks assessed per replicate. Recordings were carefully checked to exclude sample drifting. According to the criteria described by Fabbrocini et al. [104], spermatozoa with VCL > 10 µm s^−1^ were classified as motile, whereas spermatozoa with VCL > 100 µm s^−1^ were classified as rapid.

The assessed motility parameters included total motility (TM, %), progressive motility (PR, %), percentage of rapid spermatozoa (RAPID, %), and sperm velocity descriptors, including curvilinear velocity (VCL, μm s^−1^), average path velocity (VAP, μm s^−1^), and straight-line velocity (VSL, μm s^−1^). Additional trajectory descriptors included straightness (STR, %), linearity (LIN, %), wobble coefficient (WOB, %), amplitude of lateral head displacement (ALH, μm), and beat-cross frequency (BCF, Hz).

**Figure 1:**
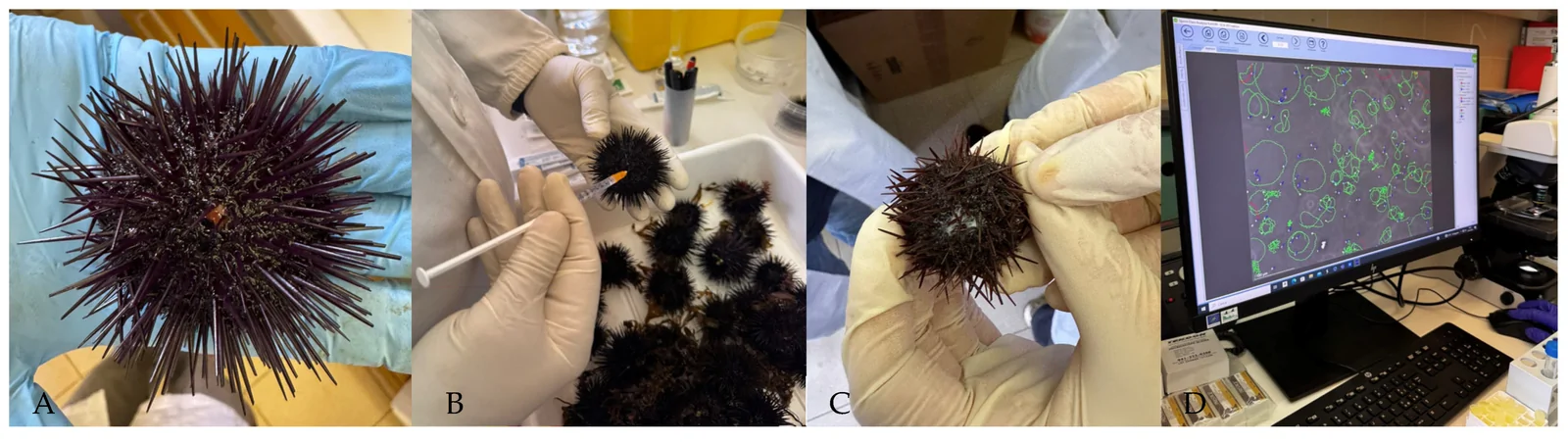
Representative steps of sperm collection and quality assessment performed during the preliminary activities of the AVATIM project. (**A**) Adult specimen of *P. lividus* selected for reproductive assessment; (**B**) induction of gamete release through the intracoelomic injection of 1 mL of 0.5 M KCl; (**C**) Collection of undiluted (“dry”) semen from a male *Paracentrotus lividus* following KCl-induced spawning. (**D**) Evaluation of sperm motility and kinematic parameters by Computer-Assisted Sperm Analysis (CASA) using the Sperm Class Analyser^®^ software.

Sperm membrane integrity was evaluated by flow cytometry using a Muse^®^ Cell Analyzer (Luminex Corporation, Austin, TX, USA), following the manufacturer’s protocol. Semen samples were initially diluted in phosphate-buffered saline (PBS) to obtain a final concentration ranging from 1 × 105 to 1 × 107 spermatozoa mL^−1^. Subsequently, 20 μL of diluted semen was added to 780 μL of Muse^®^ Count & Viability Kit reagent (dilution factor 1:40) and incubated for 5 min in the dark at room temperature. Samples were then analysed, and results were reported as nucleated cell populations and viability percentages. The main steps of specimen handling, induction of gamete release, and computer-assisted sperm analysis performed during the preliminary AVATIM activities are shown in Figure 1.

### 7.2. Baseline Sperm Quality of Paracentrotus lividus

The preliminary reproductive and sperm quality assessments generated a baseline dataset including ejaculate volume, sperm concentration, motility, kinematic descriptors, and sperm viability (Table 4). These baseline values provide an initial methodological framework for subsequent cryopreservation experiments and germplasm biobanking research in *P. lividus*.

Overall, the values obtained indicate high fresh-sperm motility and membrane integrity under the experimental conditions adopted. However, these parameters alone cannot predict cryotolerance or post-thaw reproductive performance, which remain to be experimentally evaluated. Future AVATIM activities should therefore evaluate post-thaw motility, fertilization capacity, larval development, and inter-population variability.

These findings are consistent with previous reports in the literature [101,104]. Although methodological differences among studies may influence the absolute values obtained, the available evidence suggests that the sperm quality of this species can be maintained at levels suitable for reproductive management and the long-term conservation of genetic resources.

In several aquatic species, sperm motility and velocity parameters have been associated with fertilization and hatching success, although the strength of these relationships may vary among species and according to the experimental conditions and sperm-to-egg ratio employed [122,123,124]. For this reason, computer-assisted sperm analysis (CASA) systems are increasingly used to provide rapid, objective, and quantitative assessments of sperm motility and kinematics, reducing the operator-dependent variability associated with conventional subjective observations [125,126].

Among the parameters quantified by CASA systems, total motility (TM, %), progressive motility (PR, %), curvilinear velocity (VCL, μm s^−1^), straight-line velocity (VSL, μm s^−1^), and linearity (LIN, %) are commonly used to characterize sperm quality and may be associated with fertilizing ability in some aquatic species [122,124]. Curvilinear velocity represents the average velocity of a spermatozoon along its actual trajectory, whereas straight-line velocity is calculated from the straight-line distance between the first and last recorded positions divided by the tracking time. Linearity, calculated as VSL/VCL × 100, describes the rectilinearity of the sperm trajectory.

Additional kinematic descriptors provide complementary information on sperm swimming patterns [122,127]. Average path velocity (VAP, μm s^−1^) represents the velocity along a computationally smoothed trajectory. Wobble (WOB, calculated as VAP/VCL × 100) describes the oscillation of the actual trajectory around the average path, whereas straightness (STR, calculated as VSL/VAP × 100) indicates the linearity of the average path. The amplitude of lateral head displacement (ALH, μm) quantifies the lateral movement of the sperm head around the average path, while beat-cross frequency (BCF, Hz) represents the frequency with which the actual trajectory crosses the average path.

### 7.3. Physiological Basis of Sperm Motility in Sea Urchins

Sea urchin sperm motility represents one of the most extensively investigated aspects of echinoid reproductive biology and constitutes a model system for understanding the molecular mechanisms regulating sperm activation and flagellar movement [128,129]. Within the gonads, spermatozoa are maintained in a quiescent and metabolically inactive state, which is essential for preserving intracellular ATP reserves prior to gamete release. This condition is mainly maintained by the relatively acidic intracellular environment and elevated CO_2_ pressure, which inhibit the dynein ATPases responsible for flagellar movement [129,130,131].

Following release into seawater, changes in the physicochemical properties of the external medium, including osmolarity, CO_2_ concentration, and ionic composition, induce rapid intracellular alkalinization through the activation of Na^+^/H^+^ exchangers. The increase in intracellular pH stimulates mitochondrial respiration, ATP production, and soluble adenylate cyclase activation, resulting in increased intracellular cyclic AMP (cAMP) levels. At the same time, Ca^2+^ influx and Ca^2+^- and cAMP-dependent signaling pathways promote axonemal dynein activation, triggering flagellar beating and sperm motility [128,131,132].

From a kinematic perspective, sea urchin spermatozoa typically exhibit circular or helical trajectories with diameters of approximately 50 μm, representing an efficient search strategy in the three-dimensional marine environment [133,134]. Variations in intracellular Ca^2+^ concentration modulate flagellar beat asymmetry and allow spermatozoa to rapidly change swimming direction in response to egg-derived chemoattractant peptides, thereby facilitating chemotaxis and maximizing fertilization success [128,132,134,135].

Unlike most teleost fishes, in which sperm motility is generally exhausted within minutes after activation due to osmotic and energetic constraints, echinoid spermatozoa can maintain high percentages of motile cells and relatively stable kinematic parameters even after prolonged incubation in seawater. In *P. lividus*, the proportion of motile spermatozoa remains high after extended incubation, with only minor variations in certain kinematic parameters [136,137,138,139]. This persistence reflects the efficiency of ionic regulatory mechanisms and energy metabolism in echinoid spermatozoa and represents an experimental advantage, as it broadens the temporal window available for sample manipulation, CASA standardization, and post-thaw sperm quality assessment.

### 7.4. Sperm Viability and Implications for Cryopreservation

In addition to motility, plasma membrane integrity represents one of the principal indicators of semen quality and is widely used to assess sperm viability and the suitability of gametes for cryopreservation procedures. The plasma membrane is a highly specialized and dynamic structure that contributes to cellular homeostasis by regulating the selective exchange of ions, water, and metabolites between the intracellular and extracellular environments. Its integrity is essential not only for cell survival but also for maintaining the physiological processes involved in sperm activation, motility, chemotaxis, and fertilization [128,140,141].

Sperm viability is generally defined as the proportion of spermatozoa that retain an intact plasma membrane [140,142]. Loss of membrane integrity alters selective permeability and ion transport and may be associated with impaired mitochondrial activity, reduced ATP availability, and the progressive loss of sperm motility and functionality. In this context, membrane integrity provides information complementary to motility assessment, since motile spermatozoa do not necessarily retain full structural and functional competence [140,142].

The plasma membrane also represents one of the principal targets of cryopreservation-induced damage. During freezing and thawing, spermatozoa are exposed to osmotic stress, cellular dehydration, extracellular ice formation, and alterations in membrane lipid organization. At the same time, the production of reactive oxygen species may promote lipid peroxidation and protein oxidation, potentially impairing ion channels and transport systems involved in sperm activation and the maintenance of motility. Together, these processes may reduce post-thaw sperm viability and functionality, making the preservation of membrane integrity a central objective of cryopreservation protocols [115,143,144].

Since no single parameter is sufficient to comprehensively describe semen quality, sperm quality assessment generally integrates viability with analyses of motility, mitochondrial activity, acrosomal integrity and function, DNA integrity, and fertilizing ability [140,145].

Of particular importance, especially in the context of cryopreservation, is the preservation of acrosomal structure and function, which should be considered alongside sperm motility and plasma membrane integrity when assessing fertilizing competence. In *P. lividus*, Scalisi et al. [146] evaluated acrosomal integrity together with sperm viability and fertilization outcomes. Ultrastructural studies have shown that *P. lividus* spermatozoa possess an acrosomal vesicle located at the apical region of the sperm head [147]. During the acrosome reaction, exocytosis of the acrosomal vesicle leads to the formation of the acrosomal process and the exposure of bindin, a sperm protein involved in sperm–egg interaction and fertilization [148].

This multiparametric approach provides a more complete characterization of sperm functionality than motility assessment alone and may improve the evaluation of cryopreservation outcomes [140,145].

In the present study, sperm viability was assessed using the Muse^®^ Cell Analyzer, an automated fluorescence-based cytometric platform that enables rapid and objective quantification of viable and non-viable cell populations using membrane-integrity-sensitive fluorescent dyes. Compared with traditional microscopic evaluations, cytometric approaches can reduce operator-dependent variability and allow the analysis of a large number of cells within a relatively short period of time [149,150]. The viability values observed in fresh *P. lividus* samples, which exceeded 90%, indicate that a high proportion of the analyzed spermatozoa retained an intact plasma membrane. These findings were also consistent with the favorable motility values recorded in the same samples.

The integration of CASA-derived motility data and cytometric viability measurements enabled the definition of a baseline set of sperm quality parameters for *P. lividus*, providing a useful reference framework for the subsequent optimization of cryopreservation protocols within the AVATIM project. Overall, these findings support the importance of standardized multiparametric sperm quality assessment for the development of effective cryopreservation strategies and the future establishment of aquatic germplasm biobanks.

## 8. Future Perspectives

Future research on *P. lividus* should prioritize the standardization of reproductive and sperm-quality assessment methods, particularly CASA procedures, to improve methodological comparability and inter-laboratory reproducibility. In parallel, cryopreservation protocols require further optimization and validation, including freezing and thawing conditions, post-thaw sperm functionality, fertilization success, and subsequent embryonic and larval development. These endpoints are essential to determine the actual value of cryopreserved sperm for reproductive management and germplasm conservation.

The establishment of representative germplasm collections will also require appropriate genetic sampling strategies to capture intra- and inter-population diversity and to avoid overrepresentation of a limited number of donors. Genetic and genomic characterization may therefore support donor selection and long-term management of cryobank collections [55]. In addition, dedicated studies are needed to determine the relevance and appropriate scope of microbiological and biosafety monitoring in *P. lividus* semen collection, processing, and cryostorage.

From an aquaculture perspective, future studies should evaluate how sperm cryobanking can be integrated with hatchery reproductive management without reducing the need for female broodstock, viable oocytes, and reliable juvenile production. At the production level, further technical and economic validation of *P. lividus* echinoculture and its integration into IMTA systems is required, particularly at pilot and larger scales, to assess production efficiency, operational costs, biological performance, and practical scalability [28,151]. Overall, these research priorities will help define the realistic contribution of reproductive biotechnology and germplasm biobanking to the sustainable aquaculture and conservation-oriented management of *P. lividus*.

## 9. Conclusions

*Paracentrotus lividus* is a key species for Mediterranean coastal ecosystems and for the development of sustainable aquaculture practices. Through its role as a benthic herbivore, the sea urchin regulates macroalgal growth and epiphytic communities, contributing to habitat balance, biodiversity maintenance, and ecosystem resilience.

The progressive decline of natural populations, driven by a combination of overexploitation, climate change, and environmental degradation, makes it necessary to adopt integrated approaches that combine biodiversity conservation with the productive valorisation of the species. In this context, Integrated Multi-Trophic Aquaculture (IMTA) and reproductive biotechnologies emerge as complementary tools for promoting a more sustainable management of marine resources. In particular, germplasm cryopreservation and the development of cryobanks represent promising solutions for safeguarding genetic diversity, supporting restocking programs, and improving aquaculture production. The activities initiated within the AVATIM project include the characterization of sperm quality as a prerequisite for the establishment of a sperm cryobank, which will be implemented over the course of the project. Overall, the AVATIM initiative explores an integrated approach in which aquaculture, reproductive biotechnology, and genetic-resource conservation are considered within the same research framework. These approaches may support conservation-oriented management of *P. lividus* when integrated with appropriate hatchery practices, genetic management, and in situ conservation measures; however, their broader contribution to population recovery and ecosystem restoration requires further empirical validation.

## Figures and Tables

**Table 1:** Search strings used in Scopus and Web of Science Core Collection and the corresponding number of records retrieved.

Thematic Area	Search String	Scopus	Web of Science
Ecology and conservation	“*Paracentrotus lividus*” AND (ecology OR conservation)	158	1827
Fisheries, aquaculture, and IMTA	“*Paracentrotus lividus*” AND (fishery OR aquaculture OR IMTA)	174	740
Cryopreservation	“*Paracentrotus lividus*” AND cryopreservation	16	38
Germplasm banking and biobanking	(“germplasm banking” OR biobanking) AND aquatic	7	58
Microbiological quality and microbiota	“*Paracentrotus lividus*” AND (microbiota OR microbiome OR “microbial community” OR “microbiological quality” OR pathogen* OR “food safety”)	49	54

**Table 2:** Major bottlenecks limiting the development of *P. lividus* aquaculture.

Bottleneck	Main Consequence	Research Priority	Reference
Slow growth	Long production cycles and high costs	Genetic selection and optimized nutrition	[61,62]
Dependence on wild broodstock	Limited sustainability and genetic management	Closing the life cycle in captivity	[63]
Larval sensitivity	High mortality and variable juvenile output	Hatchery optimization and microbiome management	[63,64]
Feed formulation	Variable gonad quality	Sustainable functional feeds	[65,66,67,68,69]

**Table 4:** Baseline sperm quality parameters of *Paracentrotus lividus* obtained during the preliminary phase of the AVATIM project.

Parameter	Mean Value ± SD
Ejaculate volume (mL)	0.45 ± 0.05
Sperm concentration (×10^9^ sperm mL)	19.80 ± 3.68
Total motility (%)	87.70 ± 7.70
Progressive motility (%)	62.73 ± 17.12
Rapid motility (%)	58.19 ± 18.89
VCL (μm s^−1^)	126.63 ± 30.84
VAP (μm s^−1^)	106.26 ± 29.80
VSL (μm s^−1^)	78.28 ± 25.44
STR (%)	69.24 ± 10.55
LIN (%)	56.73 ± 11.48
WOB (%)	76.37 ± 7.43
ALH (μm)	1.76 ± 0.23
BCF (Hz)	13.62 ± 6.04
Viability (%)	95.1 ± 1.32

VCL: curvilinear velocity; VAP: average path velocity; VSL: straight-line velocity; STR (VSL/VAP × 100): straightness; LIN (VSL/VCL × 100): linearity; WOB (VAP/VCL × 100): wobble; ALH: amplitude of lateral head displacement; BCF: beat cross frequency.

## Data Availability

The data presented in this study are available from the corresponding author upon reasonable request. The experimental data reported herein were generated during the preliminary phase of the AVATIM project.
